# Blood levels of neurofilament light are associated with disease progression in a mouse model of spinocerebellar ataxia type 3

**DOI:** 10.1242/dmm.050144

**Published:** 2023-09-04

**Authors:** David Mengel, Isabel G. Wellik, Kristen H. Schuster, Sabrina I. Jarrah, Madeleine Wacker, Naila S. Ashraf, Gülin Öz, Matthis Synofzik, Maria do Carmo Costa, Hayley S. McLoughlin

**Affiliations:** ^1^ Research Division Translational Genomics of Neurodegenerative Diseases, Hertie-Institute for Clinical Brain Research and Center of Neurology, University of Tübingen, Tübingen 72076, Germany; ^2^ German Center for Neurodegenerative Diseases (DZNE), University of Tübingen, Tübingen 72076, Germany; ^3^ Department of Neurology, University of Michigan, Ann Arbor, MI 48109-2200, USA; ^4^ Center for Magnetic Resonance Research, Department of Radiology, Medical School, University of Minnesota, Minneapolis, MN 55455, USA

**Keywords:** Ataxia, *ATXN3*, Blood biomarker, Neurochemical, Neurodegeneration, Polyglutamine

## Abstract

Increased neurofilament light (NfL; NEFL) protein in biofluids is reflective of neurodegeneration and has gained interest as a biomarker across neurodegenerative diseases. In spinocerebellar ataxia type 3 (SCA3), the most common dominantly inherited ataxia, patients exhibit progressive NfL increases in peripheral blood when becoming symptomatic, and NfL remains stably elevated throughout further disease course. However, progressive NfL changes are not yet validated in relevant preclinical SCA3 animal models, hindering its application as a biomarker during therapeutic development. We used ultra-sensitive single-molecule array (Simoa) to measure blood NfL over disease progression in YACQ84 mice, a model of SCA3, assessing relationships with measures of disease severity including age, CAG repeat size and magnetic resonance spectroscopy. YACQ84 mice exhibited plasma NfL increases that were concomitant with ataxia-related motor deficits as well as increased serum NfL, which correlated with previously established neurometabolite abnormalities, two relevant measures of disease in patients with SCA3. Our findings establish the progression of NfL increases in the preclinical YACQ84 mouse, further supporting the utility of blood NfL as a peripheral neurodegeneration biomarker and informing on coinciding timelines of different measures of SCA3 pathogenesis.

## Teaser

**Summary:** Peripheral blood of YACQ84 mice, a model of spinocerebellar ataxia type 3, exhibits increased neuronal-specific NfL, directly associated with disease progression, providing a biomarker to interrogate in preclinical therapeutic studies.

## INTRODUCTION

Neurofilament light chain (NfL; NEFL) is the most abundant subunit of neurofilaments, which are neuron-specific cytoskeletal proteins with crucial roles in the structural integrity, electrical signal conduction and synaptic function of neurons (Gaetani et al., 2019). Of the heavy, medium and light chains that compose neurofilaments, NfL is the most soluble, and can be actively or passively released by cells and detected in biofluids including the cerebrospinal fluid (CSF) (Wilke et al., 2020b; Li et al., 2019; Peng et al., 2022; Garcia-Moreno et al., 2022; Jansen-West et al., 2022; Johnson et al., 2021), blood plasma (Wilke et al., 2020b; Johnson et al., 2018; Peng et al., 2022; Garcia-Moreno et al., 2022; Jansen-West et al., 2022; Coarelli et al., 2021; Byrne et al., 2017) and serum (Wilke et al., 2018, 2020b; Li et al., 2019; Peng et al., 2022; Soylu-Kucharz et al., 2017). NfL has been shown to be constantly released from neurons to CSF and blood biofluids at low levels in an age-dependent manner, with increased release associated with aging in healthy individuals (Gaetani et al., 2019; Khalil et al., 2020). Owing to a key role in providing structural support during radial axon growth, NfL is especially highly expressed in large, myelinated axons and is additionally released at high levels in response to axonal damage (Peng et al., 2022; Gaetani et al., 2019). Elevated levels of NfL in biofluids are considered a strong biomarker for the rate of neuronal turnover, e.g. due to death and degeneration (Wilke et al., 2022b; Benatar et al., 2023; Wilke et al., 2022a,b); therefore, NfL has been investigated as a biomarker in individuals affected by neurodegenerative diseases.

Increased biofluid NfL has been observed in patients with Alzheimer's disease (Molinuevo et al., 2018; Benedet et al., 2020; Preische et al., 2019), Parkinson's disease (Mattsson et al., 2019; Hall et al., 2012; Wilke et al., 2020a), parkinsonian disorders (Hall et al., 2012; Hansson et al., 2017), multiple system atrophy – cerebellar subtype (Wilke et al., 2018), sporadic adult-onset ataxia (Wilke et al., 2018), Friedreich's ataxia (Peng et al., 2022), amyotrophic lateral sclerosis (Weydt et al., 2016; Gaiani et al., 2017), multiple sclerosis (Kuhle et al., 2019; Disanto et al., 2017), Charcot-Maire-Tooth disease (Sandelius et al., 2018), frontotemporal dementia (Rohrer et al., 2016; Wilke et al., 2022b) and ataxia telangiectasia (Peng et al., 2022), establishing NfL as a broad biomarker of neurodegeneration. Additionally, there has been increasing evidence of altered NfL in trinucleotide repeat expansion disorders, notably the polyglutamine (polyQ) diseases. These neurodegenerative diseases differ in the implicated gene but share a common mechanism of protein gain of toxic function caused by a polyQ-expansion from a CAG repeat in the disease gene. Of the polyQ diseases, biofluid NfL has been studied in patients with Huntington's disease (Johnson et al., 2018, 2021; Byrne et al., 2017) and multiple spinocerebellar ataxias (SCAs), including SCA1 (Wilke et al., 2018, 2022a; Coarelli et al., 2021; Peng et al., 2022), SCA2 (Peng et al., 2022; Coarelli et al., 2021), SCA3 (Wilke et al., 2018, 2020b; Peng et al., 2022; Garcia-Moreno et al., 2022; Coarelli et al., 2021; Prudencio et al., 2020; Li et al., 2019), SCA6 (Peng et al., 2022; Wilke et al., 2018) and SCA7 (Peng et al., 2022; Coarelli et al., 2021).

SCA3, caused by an expanded CAG repeat in the *ATXN3* gene (Kawaguchi et al., 1994), is one of the most common dominantly inherited ataxias worldwide (Gaspar et al., 2001; Schols et al., 2004) and is characterized by cerebellar degeneration and progressive ataxia. With recent advances in therapeutic development for treatment of SCA3 (Costa, 2020; Neves-Carvalho et al., 2020), there has been increased interest in responsive biomarkers informative of disease progression and efficacy of therapeutic intervention. In particular, NfL has been evidenced to hold notable value as a biomarker for SCA3. Patients with SCA3 exhibit significantly higher serum, plasma and CSF NfL levels than those of unaffected controls (Wilke et al., 2018, 2020b; Li et al., 2019; Peng et al., 2022; Garcia-Moreno et al., 2022; Coarelli et al., 2021; Prudencio et al., 2020; Faber et al., 2023 preprint). Remarkably, SCA3 carriers in pre-ataxic stages also show increased NfL in biofluids prior to age of onset of clinical ataxia presentation (Wilke et al., 2020b; Li et al., 2019). Specifically, pre-ataxic patients demonstrate significantly higher serum NfL than that of unaffected controls more than 10 years prior to ataxia onset (Faber et al., 2023 preprint). Furthermore, biofluid NfL levels in patients with SCA3 have been found to correlate with various measures of disease progression and severity, including age (Wilke et al., 2020b; Li et al., 2019; Coarelli et al., 2021), CAG repeat size (Wilke et al., 2020b; Coarelli et al., 2021), Scale for Assessment and Rating of Ataxia (SARA) and International Cooperating Ataxia Rating Scale (ICARS) scores (Wilke et al., 2020b; Li et al., 2019; Peng et al., 2022; Garcia-Moreno et al., 2022; Coarelli et al., 2021), and volume of affected brain regions (Li et al., 2019; Peng et al., 2022; Coarelli et al., 2021).

Validation of proposed biomarkers in patients as well as in animal models is imperative for the preclinical assessment of therapeutic efficacy for a specific disease. As such, levels of biofluid NfL have additionally been studied in the context of animal models of neurodegenerative diseases. Mouse models of neurodegenerative diseases, including Huntington's disease (Soylu-Kucharz et al., 2017) and SCA3 (Wilke et al., 2020b; Garcia-Moreno et al., 2022; Jansen-West et al., 2022; Haas et al., 2022; Lin et al., 2022; Costa et al., 2020), have been shown to exhibit NfL changes similar to those seen in patients. Although levels of NfL are known to change in an age-dependent manner in patients, prior to this study, the association of blood NfL levels with molecular markers of cerebellar pathology and ataxia-like behavior abnormalities in a transgenic mouse model of SCA3 had not yet been investigated. The YACQ84 is the most frequently used preclinical mouse model of SCA3, as it expresses the full-length human mutant *ATXN3* gene harboring a polyQ-encoding CAG repeat of ∼84 trinucleotides and exhibits ataxic-like motor deficits and neuropathological signs resembling the human disease (Cemal et al., 2002; Costa et al., 2013; McLoughlin et al., 2018; Moore et al., 2017). In this study, we sought to assess the utility of NfL as a biomarker of disease progression and cerebellar pathology in a preclinical mouse model of SCA3 by characterizing changes in blood NfL levels throughout disease progression in YACQ84 mice.

## RESULTS

### Aged homozygous YACQ84 transgenic mice show increased levels of serum NfL

Because clinical trials for SCA3 are routinely preceded by preclinical assays and interventions conducted in mouse models of this disease, it is essential that these models show biofluid biomarkers and biomarker changes similar to those of patients with SCA3. Hence, we sought to evaluate whether aged SCA3 YACQ84 transgenic mice expressing the full-length human *ATXN3* disease gene (Cemal et al., 2002), frequently used in preclinical trials for SCA3 (Rodriguez-Lebron et al., 2013; Ashraf et al., 2019; McLoughlin et al., 2018; Moore et al., 2017; Costa et al., 2013), replicate the increased blood NfL levels observed in patients with SCA3 (Wilke et al., 2018, 2020b; Li et al., 2019; Peng et al., 2022; Garcia-Moreno et al., 2022; Coarelli et al., 2021).

**Fig. 1.**
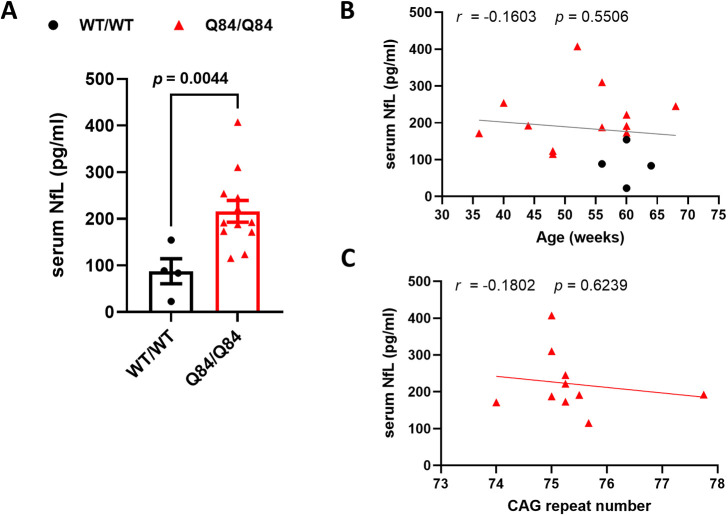
**Serum NfL levels are increased in aged Q84/Q84 mice**. (A) Serum NfL levels (average of technical duplicates) of 36- to 68-week-old homozygous Q84/Q84 mice (*n*=12) are more than double those of their 50- to 64-week-old WT/WT littermates (*n*=4). Each individual data point indicates a single mouse. Bars represent mean±s.e.m. The displayed *P*-value between mouse groups was obtained by linear regression adjusted for age (*F*=0.0132). Age did not have an effect on the observed differences in serum NfL between the mouse groups (*P*=0.8860; *F*=0.02143). (B) Serum NfL levels are not correlated with mouse age. (C) Serum NfL levels of Q84/Q84 mice are not correlated with the CAG repeat size. Associations were assessed using Spearman's rank correlation. NfL, neurofilament light; *r*, Spearman *r*; WT/WT, wild type. *P<*0.05 was considered significant (bold).

Using established ultra-sensitive single-molecule array (Simoa)-based protocols (Wilke et al., 2020b), we measured blood serum NfL levels and correlated them with previously measured cerebellar neurochemical and pathological alterations (Costa et al., 2020). We used previously collected serum samples from the time of death immediately following magnetic resonance spectroscopy (MRS) scanning from homozygous YACQ84 (Q84/Q84) mice (average age, 52.3 weeks) and their non-transgenic wild-type (WT/WT) littermates (average age, 60.0 weeks). Aged Q84/Q84 mice replicated the findings in SCA3 patients (Wilke et al., 2020b) by showing, on average, more than double [216.1±23.40 (s.e.m.) pg/ml] the serum NfL levels displayed by WT/WT mice (87.3±26.87 pg/ml) (Fig. 1A). NfL levels did not correlate with age in the two groups of mice (Fig. 1B) or with the CAG repeat size in Q84/Q84 mice (Fig. 1C). However, because serum NfL levels are known to increase with age in unaffected individuals (Byrne et al., 2017) as well as with age in SCA3 (Wilke et al., 2020b), the comparison of NfL levels between mouse groups was corrected for this co-variable.

### Serum NfL levels in homozygous YACQ84 mice correlate with select neurochemical markers of cerebellar damage

We have previously reported that decreased levels of *N*-acetylaspartate (NAA), myo-inositol (myo-Ins) and total choline (tCho), and increased levels of glutamine (Gln) are potential MRS biomarkers of cerebellar neuronal/axonal injury, demyelination and astrogliosis in Q84/Q84 mice (Costa et al., 2020). To determine whether increased blood serum NfL levels in Q84/Q84 mice are associated with cerebellar damage, we correlated individual serum NfL levels with the previously acquired neurochemical levels in the cerebellar vermis of the same mice (Costa et al., 2020).

**Fig. 2.**
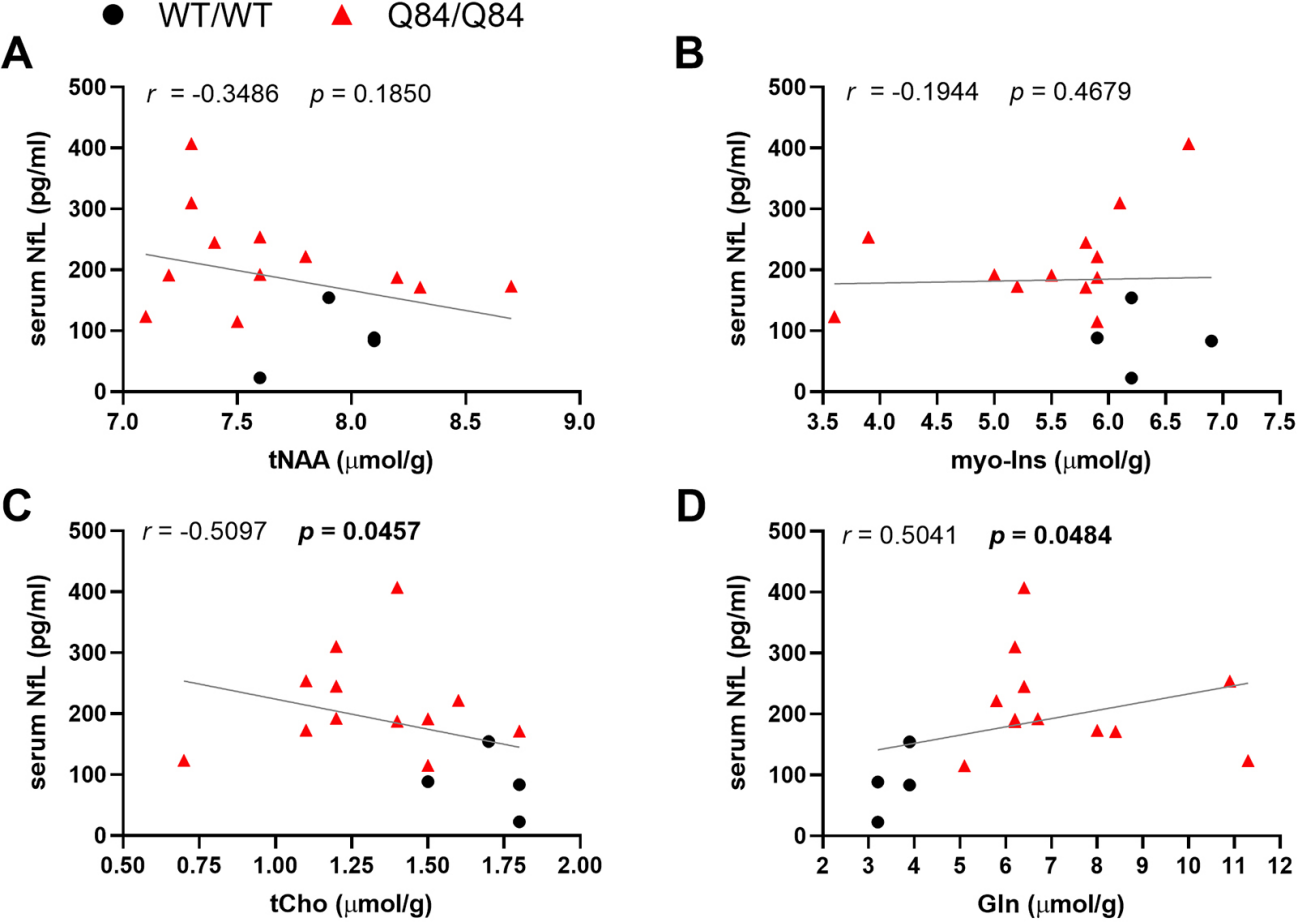
**Serum NfL levels are inversely associated with cerebellar tCho and directly associated with cerebellar Gln levels in Q84/Q84 and WT/WT littermate mice.** (A-D) Scatter plots of serum NfL levels (average of technical duplicates) with tNAA (A), myo-Ins (B), tCho (C) and Gln (D) in 36- to 68-week-old homozygous Q84/Q84 mice (*n*=12) and their 50- to 64-week-old WT/WT littermates (*n*=4). Each individual data point indicates a single mouse. Associations were assessed using Spearman's rank correlation. Gln, glutamine; myo-Ins, myo-inositol; *r*, Spearman *r*; tCho, total choline; tNAA, total *N*-acetylaspartate. *P<*0.05 was considered significant (bold).

Although blood serum NfL concentration did not correlate with levels of cerebellar total NAA (tNAA) (Fig. 2A) or myo-Ins (Fig. 2B) in aged Q84/Q84 mice, it did correlate inversely with cerebellar tCho (Fig. 2C), evidenced to be indicative of oligodendrocyte abnormalities (Costa et al., 2020), and directly with cerebellar levels of Gln (Fig. 2D), linked to astrogliosis in prior MRS studies (Öz, 2016), as previously noted. These results suggest that high levels of serum NfL could be driven by the same shared underlying factors as cerebellar axonopathy/demyelination and astrogliosis. Blood serum NfL levels were not associated with the levels of other measured neurochemicals (Fig. S1).

### Homozygous YACQ84 mice exhibit ataxia-like motor deficits and weight phenotype at early stages of disease

**Fig. 3.**
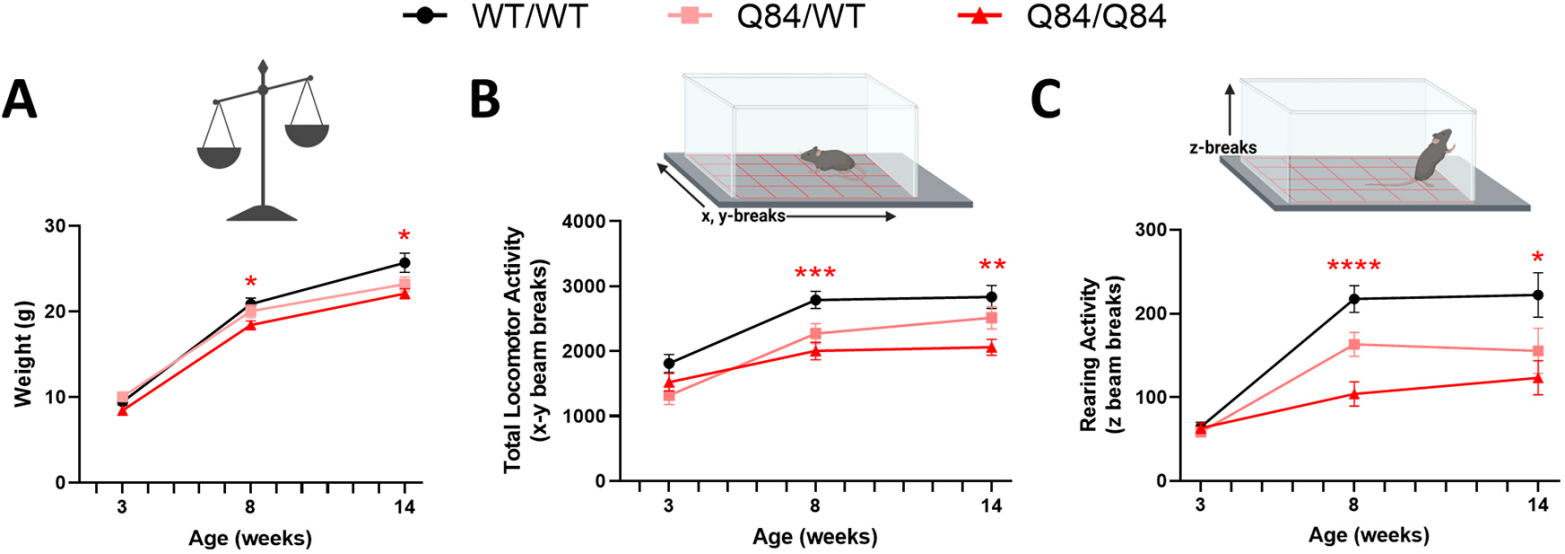
**YACQ84 mice exhibit a progressive behavioral phenotype including motor deficits.** (A-C) Weight (A) and behavioral phenotypes (B,C) were assessed in a separate cohort of mice at 3 (WT/WT *n*=20, Q84/WT *n*=16, Q84/Q84 *n*=20), 8 (WT/WT *n*=18, Q84/WT *n*=16, Q84/Q84 *n*=20) and 14 (WT/WT *n*=14, Q84/WT *n*=8, Q84/Q84 *n*=15) weeks of age. (B) Total locomotor activity assessed by *x/y*-axis beam breaks in an activity chamber. (C) Rearing assessed by *z*-axis beam breaks in an activity chamber. Each individual data point indicates a single mouse. Plots represent mean±s.e.m., with behavioral and weight measurements compared between groups by a mixed-effects model with Greenhouse–Geisser correction and a post-hoc Tukey's multiple comparisons test. Labeled *P*-values indicate post-hoc comparisons between WT/WT and Q84/Q84 mice. *P<*0.05 was considered significant (*P<0.05; **P<0.01; ***P<0.001, ****P<0.0001).

After observing elevated levels of blood NfL at late-stage disease that also correlated with select neurochemical markers of SCA3 pathology, we aimed to evaluate the progression of motor behavioral deficits and NfL levels during the disease course. Symptomatic patients with SCA3 are known to experience progressive loss of motor control with disease progression, a behavioral phenotype that is recapitulated by SCA3 mouse models (Cemal et al., 2002; Costa et al., 2013; McLoughlin et al., 2018; Moore et al., 2017). To study the temporal trajectory of ataxia-like motor deficits in our mouse model, we assessed total locomotor activity and rearing activity (open-field testing), as well as weight, in homozygous Q84/Q84, hemizygous Q84/WT and WT/WT mice at 3, 8 and 14 weeks of age (Fig. 3A-C). At 3 weeks of age, locomotor and rearing activities and weight were not significantly different between genotypes (Fig. 3). Conversely, homozygous Q84/Q84 mice exhibited significantly lower weight than that of WT/WT mice at 8 and 14 weeks of age, aligning with previous characterization of a weight phenotype in SCA3 mice (Cemal et al., 2002; Costa et al., 2013; Schuster et al., 2023) (Fig. 3A). Consistent with previous findings of symptomatic onset of motor deficits in open-field assessment at 4 weeks of age in homozygous Q84/Q84 mice (Schuster et al., 2023), homozygous Q84/Q84 mice exhibited significant motor deficits compared to WT/WT mice at 8 and 14 weeks of age, as indicated by decreased total locomotor (Fig. 3B) and rearing activity (Fig. 3C). Hemizygous Q84/WT mice exhibited an intermediate weight and motor phenotype compared to that of WT/WT and homozygous Q84/Q84 mice (Fig. 3A-C).

### YACQ84 mice show age- and disease gene dose-dependent increases in blood NfL, coincident with the onset of ataxia-like motor deficits

**Fig. 4.**
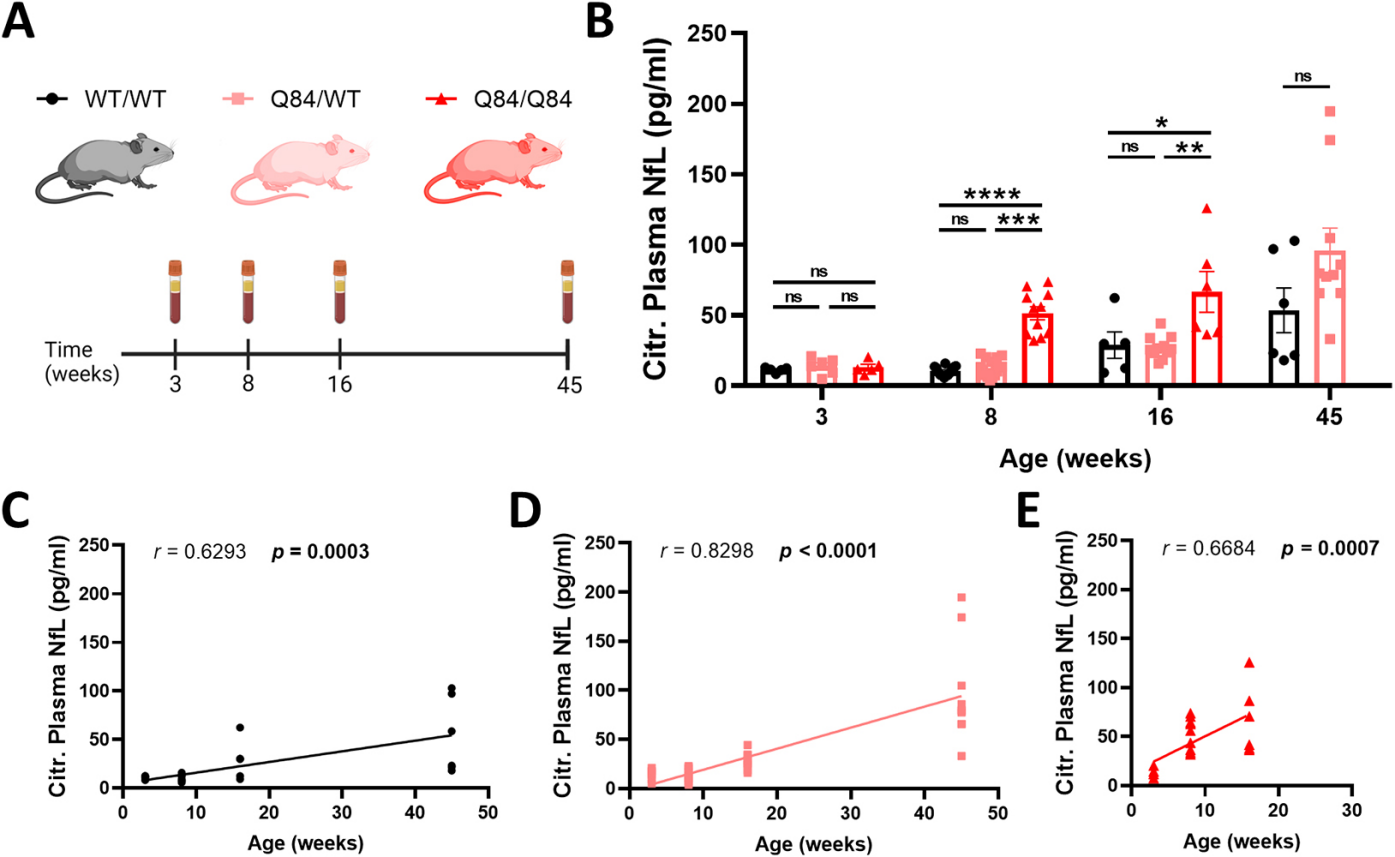
**Age- and SCA3 gene dose-dependent plasma NfL increase in YACQ84 mice.** (A) Cross-sectional blood samples from wild-type, hemizygous and homozygous SCA3 mice were collected at 3 (WT/WT *n*=5, Q84/WT *n*=6, Q84/Q84 *n*=5), 8 (WT/WT *n*=13, Q84/WT *n*=15, Q84/Q84 *n*=11), 16 (WT/WT *n*=5, Q84/WT *n*=11, Q84/Q84 *n*=6) and 45 (WT/WT *n*=6, Q84/WT *n*=10) weeks of age, and plasma was isolated. Blood was collected for each genotype at each timepoint, except for homozygous SCA3 mice at 45 weeks. (B) NfL concentration (pg/ml) in citrate-treated plasma samples was determined by ultra-sensitive single-molecule array (Simoa) (average of technical duplicates). Each individual data point indicates a single mouse. Data are represented as mean±s.e.m. Graphs represent statistical data comparison between groups, as assessed by a Kruskal–Wallis test with a post-hoc Dunn's multiple comparisons test for the 3-, 8- and 16-week groups, and by a Mann–Whitney test for the 45-week group. Labeled *P*-values indicate Mann–Whitney *P-*value and post-hoc Dunn's comparisons. *P*<0.05 was considered significant (ns, not significant; *P<0.05; **P<0.01; ***P<0.001, ****P<0.0001). (C-E) Correlation between plasma NfL concentration and age in WT/WT (C), hemizygous Q84/WT (D) and homozygous Q84/Q84 (E) mice was assessed by Spearman's rank correlation. Each individual data point indicates a single mouse. *P<*0.05 was considered significant (bold). Citr., citrate-treated; *r*, Spearman *r*.

After having established the onset of ataxia-like motor deficits at an age of 8 weeks, we asked whether temporal trajectories of peripheral NfL levels were correlated with the occurrence of behavioral deficits. To this end, we measured blood plasma NfL levels throughout disease progression at 3, 8, 16 and 45 weeks of age in a separate cohort of mice (Fig. 4A). At 3 weeks of age, there were no significant differences in NfL concentration between homozygous Q84/Q84, hemizygous Q84/WT and WT/WT mice (Fig. 4B). Beginning at 8 weeks of age, Q84/Q84 mice exhibited significantly increased levels of blood plasma NfL compared to those of Q84/WT and WT/WT mice (Fig. 4B). At 8 and 16 weeks, homozygous Q84/Q84 mice had significantly higher plasma NfL levels than those of hemizygous Q84/WT and WT/WT mice (Fig. 4B). Notably, the timeline of fully penetrant motor deficits in homozygous Q84/Q84 SCA3 mice aligns with this prominent disease feature of a significant NfL increase at 8 weeks in Q84/Q84 mice. Although our data demonstrate coinciding timelines of motor deficits and blood NfL increases, future studies will need to establish direct correlation in synonymous cohorts.

Following our cross-sectional analysis, we checked whether blood NfL levels also directly correlate with mouse age. Unsurprisingly, we found a linear correlation of blood plasma NfL levels with age in WT/WT (Fig. 4C), Q84/WT (Fig. 4D) and Q84/Q84 mice (Fig. 4E), demonstrating a progressive age-dependent increase in NfL concentration over time, as previously demonstrated in patients with SCA3 and unaffected individuals. Similar to aged Q84/Q84 mice (Fig. 1C), blood NfL levels did not correlate with CAG repeat size in adult Q84/Q84 mice (Fig. S2); thus, plasma NfL levels in Q84/Q84 mice were not corrected for CAG repeat size variance. These data indicate that previously established progressive motor impairments track with blood NfL increases over the course of disease, thus aligning molecular and behavioral evidence of progressive neurodegeneration in the Q84/Q84 mouse model of SCA3.

## DISCUSSION

Therapeutics for treatment of currently incurable progressive neurodegenerative diseases, including SCA3, have recently advanced to preclinical (Ashraf et al., 2019; McLoughlin et al., 2018; Nobrega et al., 2013; Costa et al., 2013; Rodriguez-Lebron et al., 2013; Kourkouta et al., 2019; Toonen et al., 2017) and clinical trials (NCT05160558, NCT03701399, NCT05490563, NCT04399265), leading to a more immediate imperative for validation of biomarkers of disease progression that can be used to asses therapeutic efficacy. However, it is first crucial to provide validation of such neurodegenerative biomarkers that span patients and relevant preclinical model systems. Therefore, we used the YACQ84 mouse, known to exhibit clinically relevant aspects of SCA3 pathogenesis (Cemal et al., 2002; Costa et al., 2013; McLoughlin et al., 2018; Moore et al., 2017), to investigate blood NfL changes throughout disease progression. The present study demonstrates that YACQ84 mice broadly mirror some of the main trends in blood NfL in patients with SCA3, specifically that aged homozygous YACQ84 transgenic mice show increased levels of blood NfL compared to those of non-diseased controls, which correlate with neurochemical signs of cerebellar damage (McLoughlin et al., 2023). We additionally describe age- and gene dose-dependent increases in blood NfL throughout disease progression, aligned with the onset of motor impairments. These findings provide information on NfL biofluid changes in the YACQ84 mouse, validating the neurodegenerative biomarkers in this frequently used SCA3 preclinical model and indicating increased opportunity for use of biomarkers in future preclinical SCA3 trials.

Studies in patients with SCA3 have demonstrated trends for NfL increases between the pre-ataxic stage and symptomatic ataxia onset (Wilke et al., 2020b; Li et al., 2019; Peng et al., 2022; Faber et al., 2023 preprint), and stably increased NfL levels during the symptomatic ataxic phase. These trends are reflective of increased neuronal turnover during the pre-ataxic phase and stably increased neuronal turnover during the symptomatic ataxic phase (Wilke et al., 2020b). Our findings in aged homozygous Q84/Q84 mice reveal synonymous trends, with significantly increased blood NfL levels at late-stage disease. Although these mice showed no correlation between age and serum NfL, this result is consistent with studies on patients with SCA3 demonstrating decreased magnitude of blood NfL increases over time (Li et al., 2019; Peng et al., 2022; Wilke et al., 2020b; Faber et al., 2023 preprint). This suggests a plateau effect, i.e. patients with SCA3 exhibit stably elevated NfL levels compared to those of unaffected individuals at late age, though there is no further acceleration of neuronal turnover over time. Thus, the lack of association between age and biofluid NfL in these mice may be explained by a NfL level plateau in late-stage disease.

Previously reported MRS cerebellar neurometabolite abnormalities (Costa et al., 2020) correlated with serum NfL in individual aged Q84/Q84 mice. In particular, increased serum NfL correlated with decreases in tCho and increases in Gln, respectively indicative of oligodendrocyte impairments and gliosis, suggesting an association between the magnitude of cellular disturbances and blood NfL. Interestingly, we found no correlation between NfL and myo-Ins, a putative marker of glial activation. Although MRS studies in patients with SCA3 (Miranda et al., 2022; Chandrasekaran et al., 2022; Adanyeguh et al., 2015) and SCA3 mice (Miranda et al., 2022) demonstrated significant increases in myo-Ins, these studies specifically looked at early-stage disease, in contrast to our aged Q84 mice. Additionally, decreased tNAA has been demonstrated in multiple neurodegenerative diseases (Adanyeguh et al., 2015), including SCA3 (Miranda et al., 2022; Chandrasekaran et al., 2022), and is classified as a marker of neuronal and axonal degeneration. A recent study in patients with SCA3 revealed notable correlations between increased serum NfL levels and neurometabolite abnormalities, specifically decreased cerebellar NAA/choline and NAA/creatine ratios, reflective of neuronal degeneration in the cerebellum (Chen et al., 2023). Intriguingly, our study found no correlation between serum NfL and cerebellar tNAA. This result could partially be because each of the biomarkers reflects a different aspect of neuronal degeneration: although tNAA levels reflect the cellular dysfunction and dendritic atrophy resulting from neurodegeneration, NfL reflects the rate of neurodegeneration (Wilke et al., 2022a; Benatar et al., 2023). Additionally, although the cerebellum is a heavily impacted region in SCA3, studies in patients and mice demonstrate additional degeneration of the brainstem, substantia nigra, spinal cord and thalamus (Rub et al., 2013), which were not assessed in this study. Thus, it is plausible that alternative regional sources of neurodegeneration are contributing to serum NfL increases, explaining the lack of correlation between serum NfL and cerebellar tNAA and myo-Ins neurochemicals.

Progressive age-dependent increases in biofluid NfL have been observed in patients with SCA3, with large increases prior to symptomatic ataxia onset (Wilke et al., 2020b; Li et al., 2019; Peng et al., 2022). Notably, unaffected controls also demonstrate progressive increases in NfL with age (Khalil et al., 2020), although to a lesser extent than patients with neurodegenerative diseases (Gaetani et al., 2019). Our results reveal YACQ84 mice to mirror these age-dependent progressive increases in blood NfL levels. Although WT/WT mice exhibited NfL increases over time, NfL increases in YACQ84 mice were accentuated in a gene dose-dependent manner, with homozygous Q84/Q84 mice showing significantly elevated NfL compared to that of heterozygous Q84/WT mice. Our previous work (Wilke et al., 2020b) showed similar trends in age-dependent biofluid NfL increases in the 304Q knock-in mouse model, which expresses 304 CAG repeats in the endogenous mouse *Atxn3* gene. The heterozygous 304Q model is characterized by symptomatic onset at 8 months, with a pre-symptomatic increase in plasma NfL at 6 months (Wilke et al., 2020b). These synonymous blood NfL increases in the endogenous system of the 304Q knock-in mouse model validate the findings of the present study, in which we demonstrate the same trends for age-dependent increase in plasma NfL in the transgenic overexpression YACQ84 mouse model with a shortened timeline defined by earlier ataxic onset. This corroborates a model in which symptomatic onset is reliant upon mutant protein expression in a dose-dependent manner. Accordingly, the YACQ84 mouse enables increased efficiency of longitudinal assessment of disease progression and therapeutic efficacy, reinforcing its utility as a preclinical model.

SCA3 is characterized by loss of motor function with disease progression, making motor deficits a foremost clinical outcome and central to relevant preclinical models. The YACQ84 mouse model faithfully recapitulates this SCA3 phenotype of progressive motor deficits (Cemal et al., 2002; Costa et al., 2013; Schuster et al., 2023), rescuable by treatment with anti-*ATXN3* antisense oligonucleotides (McLoughlin et al., 2018). Our findings show that the onset of NfL distinction between healthy mice and SCA3 diseased mice coincides with the onset of behavioral and secondary phenotypes, as evidenced by reduced weight and significant motor impairments. Although open-field activity has previously been validated as a preclinical readout of motor impairment in SCA3 mice (McLoughlin et al., 2018), future investigation of whether NfL increases are relatable to deficits in additional motor tasks would further inform the timeline of disease progression. As the heterozygous 304Q model is characterized by an increase in plasma NfL at 6 months prior to ataxia symptom onset (Wilke et al., 2020b), a deeper understanding of pre-symptomatic NfL increases in the YACQ84 SCA3 mouse model would establish opportunity for preclinical studies to use NfL as a pre-symptomatic biomarker.

Cohesively, we provide evidence of NfL as a biomarker in the YACQ84 mouse, highlighting the onset and progressive increases in NfL, as well as corresponding behavioral symptoms over time. In newly characterizing this neurodegenerative disease feature in the YACQ84 mouse, we further validate its use as a preclinical model of SCA3 and establish a basis for future studies to investigate therapeutic timelines that maximize reduction of neurodegeneration in SCA3.

## MATERIALS AND METHODS

### Mouse model and sample collection

All animal procedures were approved by the University of Michigan Institutional Animal Care and Use Committee and conducted in accordance with the United States Public Health Service's Policy on Humane Care and Use of Laboratory Animals. This study used homozygous (Q84/Q84) and hemizygous YACMJD84.2-C57BL/6 transgenic mice (Q84/WT), and non-transgenic WT/WT sex- and age-matched littermate controls. Hemizygous YACMJD84.2-C57BL/6 mice have both copies of mouse *Atxn3* in addition to one copy of human *ATXN3*. When crossed to homozygosity, homozygous YACMJD84.2-C57BL/6 mice have two copies of mouse *Atxn3* in addition to two copies of human *ATXN3.* Two cohorts of WT/WT, Q84/WT and Q84/Q84 mice were used; enrollment numbers were based on previous historical reference numbers and available data. The behavioral cohort included mice with longitudinal data collection from 3, 8 and 14 weeks of age: WT/WT *n*=20 (10 females/10 males), Q84/WT *n*=16 (8 females/8 males), Q84/Q84 *n*=20 (8 females/12 males). The NfL cohort included mice at 3 weeks [WT/WT *n*=5 (5 females), Q84/WT *n*=6 (6 females), Q84/Q84 *n*=5 (5 females)], 8 weeks [WT/WT *n*=14 (9 females/5 males), Q84/WT *n*=15 (11 females/4 males), Q84/Q84 *n*=11 (8 female/3 males)], 16 weeks [WT/WT *n*=5 (4 females/1 male), Q84/WT *n*=13 (6 females/7 males), Q84/Q84 *n*=6 (2 females/4 males)] and 45 weeks [WT/WT *n*=6 (1 female/5 males), Q84/WT *n*=10 (7 females/3 males)] of age. Animal genotypes were determined from tail biopsy DNA collected prior to weaning and confirmed post-mortem as previously described (Cemal et al., 2002; Costa et al., 2013). After genotyping, simple randomization for group assignments ensured that each mouse had an equal probability of being assigned to control or treatment group, making it an unbiased allocation process. The *ATXN3* CAG-trinucleotide repeat length was determined by gene fragmentation analysis (Laragen, Culver City, CA, USA) with *ATXN3* primers (5′-ACAGCAGCAAAAGCAGCAA-3′ and 5′-CCAAGTGCTCCTGAACTGGT-3′). CAG repeat length was calculated as (peak amplicon fragment size – 66)/3. Serum from groups of homozygous Q84/Q84 [*n*=12 (6 females/6 males), 36-68 weeks of age] and WT/WT littermate mice [*n*=4 (2 females/2 males), 56-64 weeks of age] were correlated with previously reported neurochemical concentrations measured by MRS (Costa et al., 2020). For serum collection at euthanasia, blood was collected from mice by cardiac puncture and immediately centrifuged at 15,700 ***g*** for 3 min at room temperature. The serum (supernatant) was collected in 0.6 ml Multivette 600z tubes (Sarsted, Nümbrecht, Germany) and stored at −80°C. To collect plasma, mice were euthanized and blood was collected by heart puncture with a 3.2% sodium citrate syringe. Blood was centrifuged at 4000 ***g*** for 10 mins at 4°C. Plasma was collected, aliquoted and stored at −80°C until use. Mouse blood was processed to serum or plasma to allow for different downstream analyses. Collection of all blood biofluid types from a single mouse is not feasible owing to limited amounts of available blood. In each experimental paradigm and respective figure of our paper, only one type of blood biofluid was analyzed to avoid confounding matrix effects.

### Motor assessments

Motor activity of a separate cohort of SCA3 mice relative to that of WT/WT littermates was measured in an activity chamber with a photobeam open-field apparatus (San Diego Instruments, San Diego, CA, USA). Weight was measured prior to motor assessment at each timepoint. Total locomotor activity (*x*/*y*-axis beam breaks) and rearing activity (*z*-axis beam breaks) were measured during 30-min trials. Experimenters were unaware of genotype during behavioral assessment.

### Neurofilament quantification

NfL concentrations were measured in technical duplicate using Simoa on a Simoa HD-X analyzer (Quanterix, Lexington, MA, USA). The NF-light Advantage kit (Quanterix) was used according to manufacturer's instructions. Mouse plasma and serum were re-spun at 10,000 ***g*** for 5 min at 4°C and diluted 1 in 4 or 1 in 8 with Quanterix NfL sample buffer for subsequent analysis (dilution linearity of the assay for this concentration range in mouse blood has been established by the laboratory of M.S., unpublished results). The lower limit of quantitation (LLoQ) of the assay was defined as the lowest standard (1) with a signal higher than the average signal for the blank plus 9 standard deviations, and (2) that allows a percentage recovery ≥100±20%. All measurements were above the LLoQ of the assay, and their technical replicates produced a percent coefficient of variation (%CV) of less than 15%. The LLoQ was defined as 0.48 pg/ml. The repeatability (%CV) of the NfL assay for two internal control blood samples was determined as 3.4% and 7.1%. Experimenters were unaware of genotype and phenotype prior to analysis.

### Statistical analysis

Identification of outliers for all datasets was performed post hoc using ROUT Q=1%, and outliers were removed from data analysis. For each dataset, normality of data distribution was assessed by Shapiro–Wilk test and homogeneity of variances was assessed by Bartlett's test. NfL levels in blood plasma and serum were compared between mouse genotypes by linear regression, adjusting for age in the group of aged animals, or by two-tailed Mann–Whitney test or Kruskal–Wallis test with post-hoc Dunn's multiple comparisons test in the other groups of animals. Multiplicity adjusted *P*-values are reported for multiple comparisons. Associations of plasma and serum NfL levels with previously acquired cerebellar neurochemical concentrations, the CAG repeat size and age were assessed using Spearman's rank correlations. Weight and motor activity were compared between mouse genotypes by a mixed-effects model with Greenhouse–Geisser correction, owing to unequal variance as assessed by Bartlett's test and a post-hoc Tukey's multiple comparisons test. Multiplicity adjusted *P*-values are reported for multiple comparisons. All statistical analyses were performed using Prism, version 9 (GraphPad Software, LaJolla, CA, USA). The significance threshold was set to two-sided *P*<0.05.

## Supplementary Material

10.1242/dmm.050144_sup1 Supplementary information
