# Supplementary material for: Blood levels of neurofilament light are associated with disease progression in a mouse model of spinocerebellar ataxia type 3
Source: Dis Model Mech. 2023 Sep 4;16(9):dmm050144. doi: 10.1242/dmm.050144 (PMC10499033; doi:10.1242/dmm.050144)
Supplement: Supplementary information [file dmm-16-050144-s1.pdf]

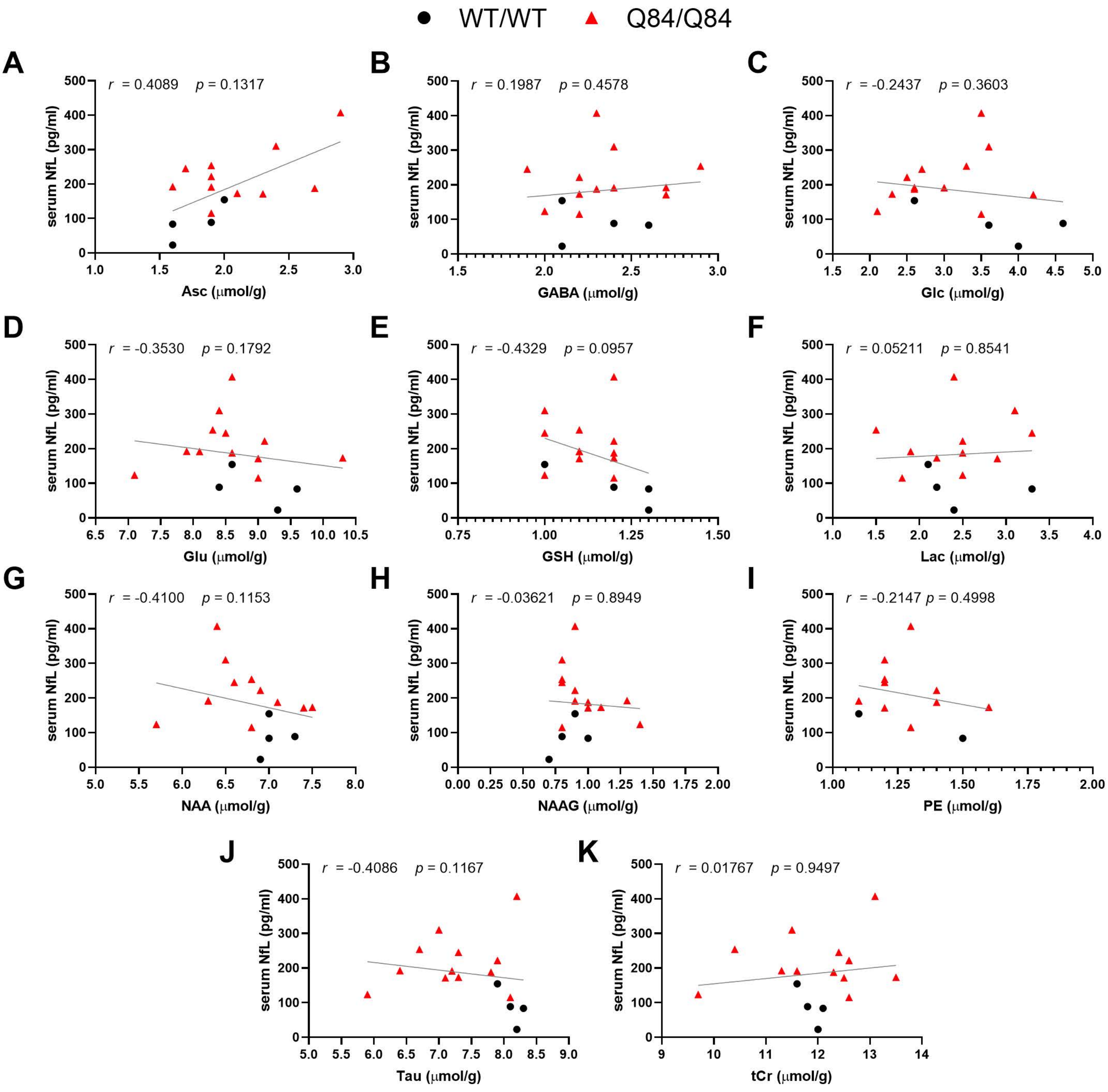

Fig. S1. Scatter plots of serum NfL levels (average of technical duplicates) do not correlate with cerebellar (A) Asc, ascorbate; (B) GABA, γ-aminobutyric acid; (C) Glc, glucose; (D) Glu, glutamate; (E) GSH, glutathione; (F) Lac, lactate; (G) NAA, *N*-acetylaspartate; (H) NAAG, *N*-acetylapartylglutamate; (I) PE, phosphoethanolamine; (J) Tau, taurine; (K) tCr, total creatine in 36-68 weeks-old homozygous Q84/Q84 (n=12) and their 50-64 weeks-old WT/WT littermates (n=4). Each individual data point indicates a single mouse. Line of best fit determined by simple linear regression. Associations performed using Spearman's rank correlation. *r*, Spearman *r*. Statistical significance was considered for $p < 0.05$.

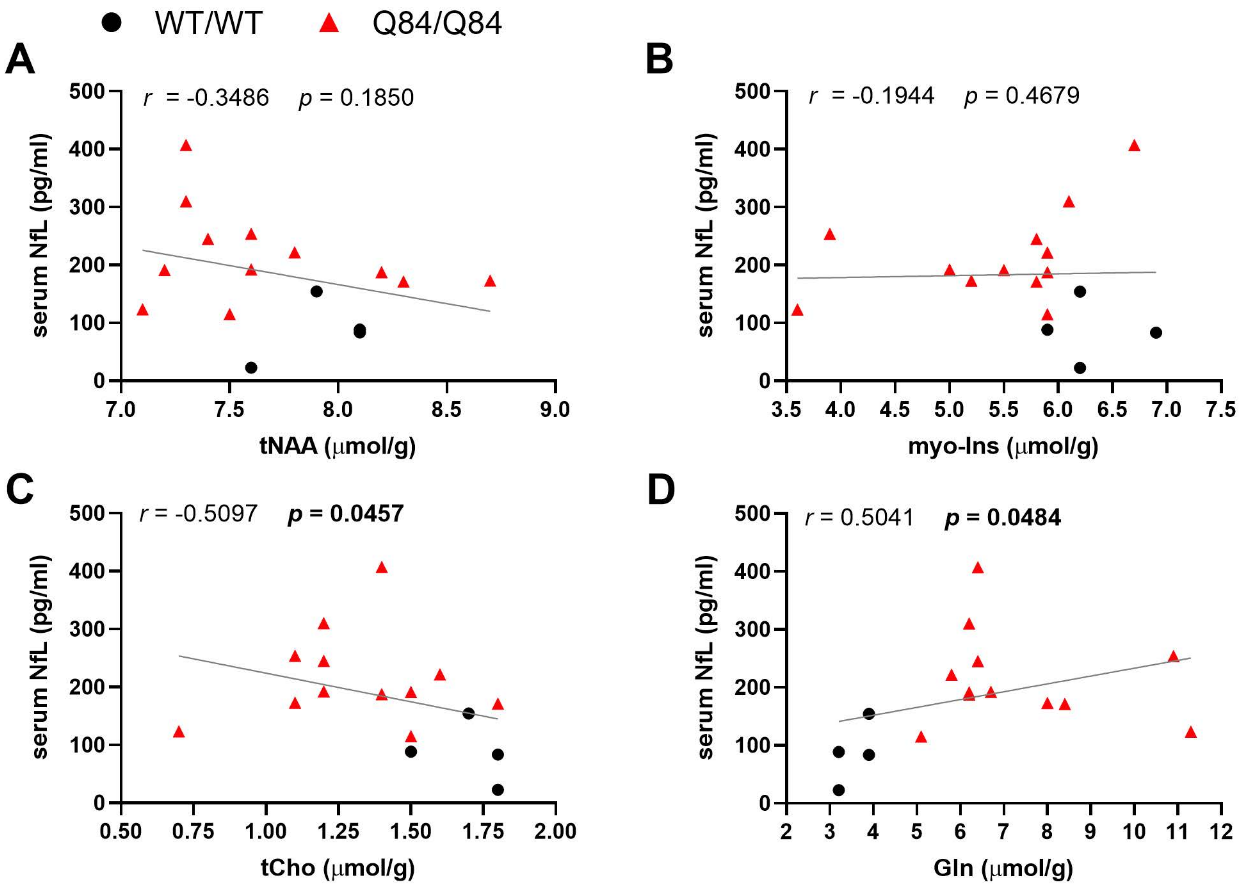

Fig. S2. Scatter plots of sodium citrate plasma NfL levels (average of technical duplicates) with CAG repeat size in (A) Q84/WT hemizygous (n=38) and (B) Q84/Q84 homozygous (n=21) mice. Each individual data point indicates a single mouse. Associations performed using Spearman's correlation. *r*, Spearman *r*. Statistical significance was considered for $p \leq 0.05$.
